# Genetic variations in the drug metabolizing enzyme, CYP2E1, among various ethnic populations of Pakistan

**DOI:** 10.7717/peerj.9721

**Published:** 2020-08-19

**Authors:** Sagheer Ahmed, Nadeem Altaf, Mahnoor Ejaz, Zaira Zulfiqar, Kholood Janjua, Dana Festila, Nicula Cristina

**Affiliations:** 1 Pharmacogenetic Laboratory, Department of Basic Medical Sciences, Shifa College of Pharmaceutical Sciences, Shifa Tameer-e-Millat University, Islamabad, Pakistan; 2 Overseas Pakistanis Foundation Boys College, Sector H-8, Islamabad, Pakistan; 3 Shifa Clinical Research Center, Shifa International Hospital, Islamabad, Pakistan; 4 University of Medicine and Pharmacy of Cluj-Napoca, Cluj-Napoca, Romania

**Keywords:** Cytochrome P450, CYP2E1, Pharmacogenomics, Pakistan

## Abstract

Genetic polymorphism in cytochrome P450 (CYP) monooxygenase genes is an important source of interindividual variability of drug response. CYP enzyme activities may change as a result of such polymorphisms which then, may affect drug metabolism. This would result in a change in the severity and frequency of adverse effects in addition to the non-responder phenomenon. CYP2E1, a member of CYP superfamily, affects the metabolism of several clinically important drugs such as halothane, paracetamol, etc. Genetic variation in *CYP2E1* is known to cause significant inter-individual differences in drug response and adverse effects. The degree of genetic variation is found to be different in different populations around the world. The frequencies of two important polymorphisms in the CYP2E1*7C, NC_000010.10:g.135340548A>G (rs2070672) and *CYP2E1*, NC_000010.10:g.135339244G>C (rs3813865), are not known in the Pakistani population. In the present investigation, 636 healthy human volunteers were screened for these two single nucleotide polymorphism. Our results indicate that about 18% (rs2070672) and 28% (rs3813865) of the Pakistani population has a genotype containing at least one low activity allele. A significant interethnic variation in the frequencies of both the polymorphisms was observed. These results suggest that pharmacogenetics screening for low activity genotypes would be a helpful tool for clinicians when they prescribe medications metabolized by CYP2E1, as a significant fraction of the Pakistani population is expected to have a variable response to these drugs.

## Introduction

Several low-molecular-weight compounds, such as N-nitrosamines, vinyl chloride, benzene, and ethanol are metabolized by the cytochrome P4502E1 (CYP2E1). This enzyme is a member of the cytochrome P450 superfamily (Arinc et al., 2007; Guengerich, Kim & Iwasaki, 1991). CYP2E1 produces more reactive oxygen species compared to many other CYP enzymes because of high redox potential (Cederbaum, 2006; Ingelman-Sundberg et al., 1993; Zhukov & Ingelman-Sundberg, 1999). Therefore, in alcohol-induced liver injury, CYP2E1 is thought to be an important source of free radicals (Cederbaum, 2006). Among the many drugs metabolized partially or completely by CYP2E1 include halothane, enflurane, isoflurane, paracetamol, ethanol, theophylline, chlorzoxazone, zopiclone, eszopiclone and verapamil (Ågerstrand, Wester & Rudén, 2009; Flockhart, 2007). In addition to metabolizing many pharmaceutical drugs, *CYP2E1* gene itself is regulated by many exogenous and endogenous substances and may substantially enhance the risk posed by many industrial and environmental chemicals (Bolt, Roos & Thier, 2003).

Selected polymorphisms in this gene are associated with a higher risk of certain diseases such as cancer (Barry et al., 2011; Zhang et al., 2009). For example, single nucleotide polymorphisms, CYP2E1*7C, NC_000010.10:g.135340548A>G (rs2070672) and *CYP2E1*, NC_000010.10:g.135339244G>C (rs3813865), are significantly associated with high altitude polycythemia risk (Xu et al., 2015), with nasopharyngeal carcinoma risk in Cantonese (Jia et al., 2009), with poorer cancer-specific survival in head & neck cancer (Hakenewerth et al., 2013). These polymorphisms are also associated with inter-individual differences in drug response and adverse effects, especially the liver injury with ethanol, and the frequency of these polymorphisms varies in different ethnic groups around the world (Bonifaz-Pena et al., 2014; Huang et al., 2012; Kim et al., 2015). As the frequencies of genetic polymorphisms are associated with interethnic differences in drug response and add significant risk for certain diseases, it is an extremely important subject to investigate. That is why frequencies of these polymorphisms in *CYP2E1* have been described for various populations.

Pakistan is a culturally diverse country, but little is known about the distribution of *CYP2E1* genetic polymorphism in this country of over 200 million people. Various parts of the country possess a unique lifestyle, diverse genetic background, dietary habits, culture, and geographical environment. There are more than 100 single nucleotide polymorphisms found in *CYP2E1* in addition to some copy number variants. Among them, rs2031920, rs3813867, rs6413432, rs6413420, rs72559710, rs55897648, rs2070673, rs3813865 and rs2070672 are well known. However, only a few might alter the enzyme activity or associated with certain diseases. Therefore, we specifically investigated samples drawn from six of Pakistan’s most populous ethnic groups located in distinct geographical locations and found out frequencies of two important polymorphisms (rs3813865 and rs2070672) and then compared them with previous findings in other populations.

## Materials and Methods

### Sample collection and DNA extraction

This study was approved by the Institutional Review Board and Ethics Committee of Shifa Tameer-e-Millat University, Islamabad, Pakistan (ref: IRB# 990-265-2018). Written informed consent forms were obtained from all participating individuals. The study cohort comprised of 636 healthy human individuals from six major ethnicities of Pakistan including Punjabis, Pathan, Sindhi, Balochi, Seraiki, and Urdu Speaking. Ethnicity was self-reported. Five ml of venous blood drawn into sterile tubes containing EDTA as an anti-coagulant was stored at 4 °C. The whole-genome DNA was isolated using Gene Jet Genomic DNA extraction Kit (Thermo Fischer, Waltham, MA, USA) and was quantified using 1% agarose gel electrophoresis. Isolated genomic DNA was stored at −20 °C until further processing.

### Genotyping

*CYP2E1* (rs3813865, G>C; rs2070672, A>G) were genotyped using Allele Refractory Mutation System-Polymerase Chain Reaction (ARMS-PCR) using a pair of outer primers and a pair of inner primers as shown in Table 1. PCR for both the SNPs was performed separately in a total reaction volume of 25 µl containing 12.5 µl of 2X Dream Taq Mastermix (Thermo Fischer, Waltham, MA, USA), 0.18 mM of both OF and OR primers, 0.36 mM of both IF and IR primers, 7.7 µl of sterile PCR water and three µl of template DNA (20–50 ng/μl). Thermal profile was as follows: initial denaturation at 95 °C for 2 min followed by 35 cycles with denaturation at 95 °C for 30 s, 30 s of primer annealing at 54 °C for rs3813865 and 58 °C for rs2070672, initial extension at 72 °C for 1 min and a final extension at 72 °C for 2 min. For visualization 12 µl of PCR product was directly loaded onto 3% agarose gel. For rs3813865, homozygous wild type GG genotype had 499 bp and 303 bp fragments, homozygous mutant CC genotype had 499 bp and 236 bp fragments and heterozygous GC genotype had three fragments; 499 bp, 303 bp, and 236 bp. Whereas for rs2070672 homozygous wild type GG genotype had 455 bp and 277 bp fragments, homozygous mutant AA genotype had 455 bp and 218 bp fragments and heterozygous GA genotype had three fragments; 455 bp, 277 bp, and 218 bp. Selected samples were sent for Sanger sequencing, and the results obtained were found to conform with results from our laboratory.

**Table 1:** Primer sequences for CYP2E1 rs3813865 and rs2070672 along with their product sizes.

	Primer ID	Primer sequence	Product size
rs3813865	OF	5′ TGA TGT TGG TTG GGC ATC TA 3′	499 bp
	OR	5′ CCT CGA GGT GAG AAC TGA CA 3′	
	IF (G allele)	5′ CTC ACC CCA CCA AAG CCT AC 3′	303 bp
	IR (C allele)	5′ CCA CAG ACT GAA ATT GAA CCC 3′	236 bp
rs3813865	OF	5′ CCA TTC ATG TGG CAG GTG GTG 3′	455 bp
	OR	5′ CCA ATG CCC TCT TGC TAC TCG TCT A 3′	
	IF (G allele)	5′ TGG AGT TCC CCG TTG TCG AG 3′	277 bp
	IR (A allele)	5′ GTC CTG CCC TTT GGC ACT CGT 3′	218 bp

### Statistical analysis

Data were compiled according to the genotype and allele frequencies estimated from the observed numbers of each specific allele. The frequency of each allele and genotype in our samples is given together with the 95% confidence interval. The confidence interval for proportions was calculated using the formula (CI = *p* ± (1.96 × SE), SE = qrt [*p*(1 − *p*)/*n*], *p* = proportion, *n* = sample size). Chi-squared test and *p* values were calculated using observed and expected frequencies as per the Hardy–Weinberg equation.

## Results

**Figure 1:**
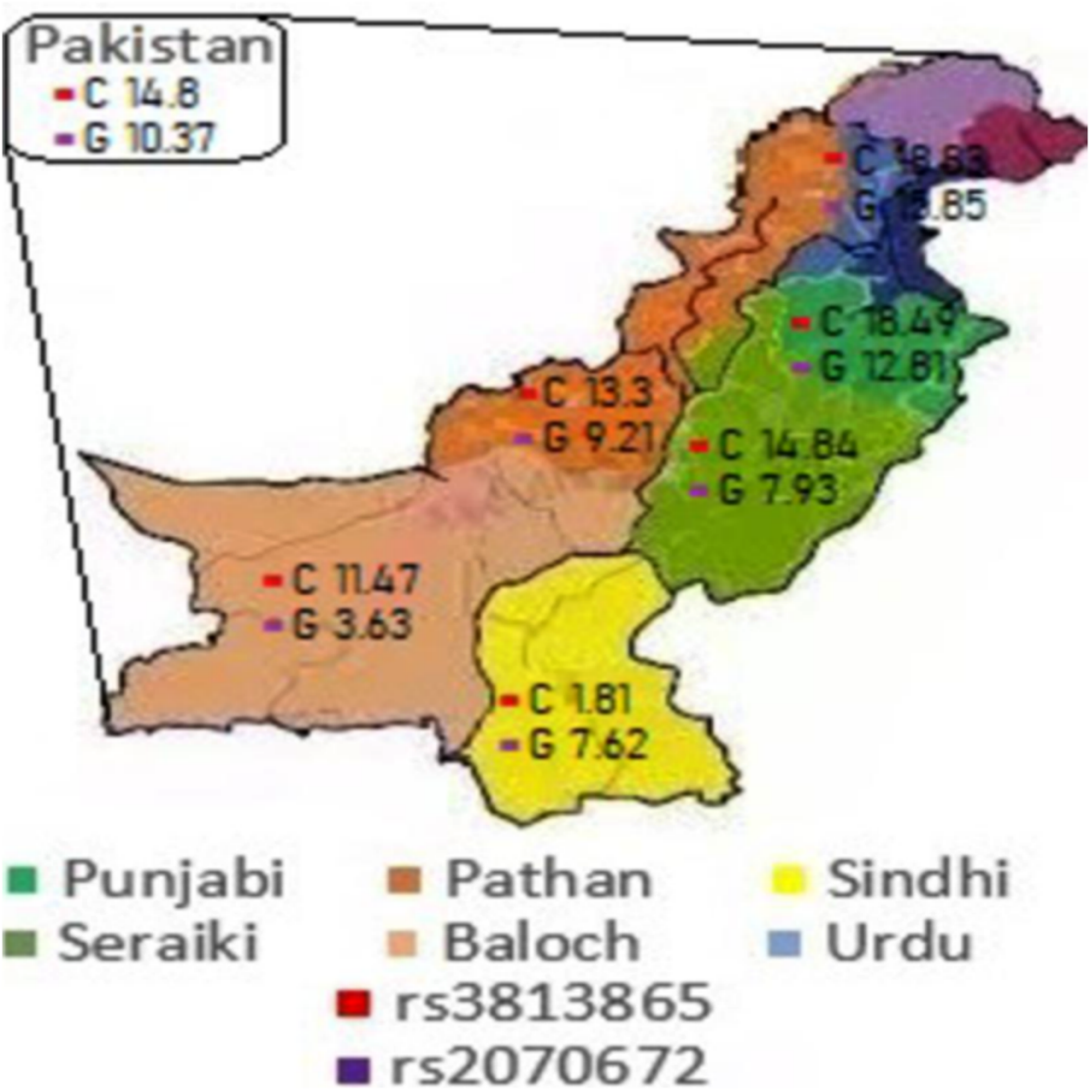
Major allele prevalence in different ethnic populations. Every color indicates an ethnic population. The red and purple quadrants inside every ethnic region represents the frequency of *CYP2E1* (rs2070672) and *CYP2E1* (rs3813865) alleles, respectively.

Frequencies of *CYP2E1* (rs2070672) alleles in the Pakistani population are shown in Table 2 while representative agarose gel image of the experiments is shown in Fig. S1. The frequency of the major allele was 89.62% and of minor allele was 10.37%. Major allele was found slightly less prevalent in Punjabi and Urdu populations at 87.18% and 84.14% respectively compared to Pathan, Sindhi, Seraiki and Baloch populations, where the prevalence of major allele was slightly higher (Fig. 1; Table 2). The frequency of AA genotype was 82.70%, AG was 13.83% and GG was 3.45% in the Pakistan population. Punjabi and Urdu populations showed a slightly lower frequency of wild type genotype at 79.83% and 78.04% respectively while Pathan, Sindhi, and Seraiki populations had a slightly higher prevalence of wild type genotype. Baloch population showed the highest frequency of wild type genotype at 92.68%. No homozygous GG genotype was found in Pathan, Seraiki and Baloch populations (Table 3).

**Table 2:** Allelic frequencies of CYP2E1 (rs2070672) invarious Pakistani ethnicities.

S. No.	Ethnicity	*N*	A% (95% CI)	G% (95% CI)
1	Pakistan	636	89.62 [87.94–91.3]	10.37 [8.69–12.05]
2	Punjabi	238	87.18 [84.18–90.18]	12.81 [9.81–15.81]
3	Pathan	107	90.65 [86.75–94.55]	9.21 [5.34–13.08]
4	Sindhi	59	92.37 [87.58–97.16]	7.62 [2.83–12.41]
5	Seraiki	68	92.64 [88.25–97.03]	7.93 [3.39–12.47]
6	Baloch	82	96.34 [93.47–99.21]	3.63 [0.77–6.49]
7	Urdu	82	84.14 [78.55–89.73]	15.85 [10.26–21.44]

**Table 3:** Observed and expected frequencies of CYP2E1 (rs2070672) genotypes.

Population	Geno	*N*	Observed frequency percentage (CI)	Expected frequency percentage by HW law	Chi squared	*P* value
Pakistani	AA	526	82.70 [79.76–85.64]	80.32	35.10	<0.05
	AG	88	13.83 [11.15–16.51]	18.58		
	GG	22	3.45 [2.03–4.87]	1.07		
Punjabi	AA	190	79.83 [74.73–84.93]	76.01	27.82	<0.05
	AG	35	14.70 [10.2–19.2]	22.33		
	GG	13	5.46 [2.57–8.35]	1.64		
Pathan	AA	87	81.30 [73.91–88.69]	82.17	1.14	>0.05
	AG	20	18.69 [11.3–26.08]	16.94		
	GG	0	0	0.87		
Urdu	AA	64	78.04 [67.08–88]	70.80	24.17	<0.05
	AG	10	12.19 [5.11–19.27]	26.66		
	GG	8	9.75 [3.33–16.17]	2.51		
Seraiki	AA	58	85.29 [76.87–93.71]	78.20	0.43	>0.05
	AG	10	14.70 [6.28–23.12]	28.87		
	GG	0	0	2.66		
Seraiki	AA	76	92.68 [87.04–98.32]	92.81	0.11	>0.05
	AG	6	7.31 [1.68–1294]	7.04		
	GG	0	0	0.13		
Sindhi	AA	51	86.44 [77.7–95.18]	85.33	1.47	>0.05
	AG	7	11.86 [3.61–20.11]	14.07		
	GG	1	1.69 [0–4.98]	0.58		

Frequencies of *CYP2E1* (rs3813865) alleles in the Pakistani population are shown in Table 4 while representative agarose gel image of the experiments is shown in Fig. S2. The frequency of minor alleles for this polymorphism was higher in the Pakistani population, compared to rs2070672, and found to be 14.8% (Table 4). In Punjabi and Urdu populations, the minor allele was found even more prevalent at 18.49% and 18.83% respectively. In Sindhi population, the frequency of minor allele was found to be the lowest at 1.81%. The frequency of the GG genotype was 72.47%, GC was 23.58% and CC was 3.93% in the Pakistan population. In Punjabi and Urdu populations, wild type genotype (GG) was slightly less prevalent at 63.47% and 67.53% respectively. Baloch population showed a higher frequency of wild type genotype at 83.60%. The highest prevalence of wild type genotype was found in the Sindhi population at 96.36%. Sindhi was also the only population in which no homozygous CC genotype was observed. All other ethnic groups showed CC genotype albeit at varying frequencies (Table 5).

**Table 4:** Allelic frequencies of CYP2E1 (rs3813865) in various Pakistani ethnicities.

S.No	Population	*N* (rs 38)	G% (95% CI)	C% (95% CI)
1	Pakistan	585	85.12 [82.34–88]	14.8 [11.92–17.68]
2	Punjabi	219	81.50 [76.36–86.64]	18.49 [13.35–23.63]
3	Pathan	109	86.69 [80.31–93.07]	13.30 [6.93–19.63]
4	Sindhi	55	98.18 [94.65–100]	1.81 [0–5.33]
5	Seraiki	64	85.15 [76.44–93.86]	14.84 [6.13–23.55]
6	Baloch	61	88.52 [80.52–96.52]	11.47 [3.47–19.47]
7	Urdu	77	81.16 [72.43–89.89]	18.83 [10.1–27.56]

**Table 5:** Observed and expected frequencies of CYP2E1 (rs3813865) genotypes.

Population	Geno	*N*	Observed frequency percentage (CI)	Expected frequency percentage by HW law	Chi Squared	*P* value
Pakistani	585					
	GG	424	72.47 [68.85–76.09]	71.01	7.08	<0.05
	GC	138	23.58 [20.14–27.02]	26.49		
	CC	23	3.93 [2.36–5.50]	2.47		
Punjabi	219					
	GG	139	63.47 [57.09–69.85]	62.76	0.40	>0.05
	GC	69	31.50 [25.35–37.65]	32.91		
	CC	11	5.02 [2.13–7.91]	4.31		
Pathan	109					
	GG	83	76.14 [68.14–84.14]	75.15	0.79	>0.05
	GC	23	21.10 [13.44–28.76]	23.06		
	CC	3	2.75 [0–5.82]	1.77		
Urdu	77					
	GG	52	67.53 [54.8–80.26]	65.88	0.89	>0.05
	GC	21	27.27 [15.17–39.37]	30.56		
	CC	4	5.19 [0–11.22]	3.54		
Seraiki	64					
	GG	46	71.87 [60.85–82.89]	72.51	0.16	>0.05
	GC	17	26.56 [15.74–37.38]	25.27		
	CC	1	1.56 (0-4.6)	2.20		
Balochi	61					
	GG	51	83.60 [74.31–92.89]	78.36	16.23	<0.05
	GC	6	9.83 [2.36–17.3]	20.30		
	CC	4	6.55 [0.34–12.76]	1.31		
Sindhi	55					
	GG	53	96.36 [91.41–100]	96.39	0.02	>0.05
	GC	2	3.63 [0–8.57]	3.56		
	CC	0	0	0.03		

## Discussion

According to its Statistics Bureau, Pakistan with an estimated population of over 210 million, is the sixth most populous country in the world (Pakistan Bureau of Statistics, 2017). The country has a young, multi-ethnic and multi-cultural society and despite being home to a huge population, pharmacogenetic studies on how its population responds to various pharmaceutical drugs are rare. The largest ethnic group in Pakistan is Punjabi, which makes up about 38.78% of the population, followed by Pashtuns (18.24%), Sindhis (14.57%), Seraikis (10.53%), Urdu speaking (7.57%) and Baloch (3.57%) (Pakistan Bureau of Statistics, 2017). These ethnic groups represent about 94% of the Pakistani population. Genetic variations in CYP genes affecting the metabolism of xenobiotics and drug response have not been investigated in these ethnic groups. Our study partly addresses this issue by reporting frequencies of two of the most important single nucleotide polymorphisms in the *CYP2E1* gene.

The frequency of rs2070672 minor allele (G) in the Pakistani population was similar to the one found in the American population (Table 6). The lowest frequency of minor allele has previously been reported from Europe (0.027). Literature search shows that in the African population, minor allele is slightly more prevalent than in the Pakistani population while East and South Asian populations have the highest frequencies of the minor allele (Genomes Project Consortium, 2015). Similar results were found for genotype frequency where the wild type genotype observed in the Pakistani population was comparable to the American population. The highest frequency of wild type genotype is reported from Europe, in which no homozygous GG genotype was found. The highest frequencies of heterozygous and homozygous GG are reported from East and South Asian populations (Genomes Project Consortium, 2015). The difference in allele and genotype frequencies between earlier reports for South Asian populations and this study may be because our study estimated frequencies in six different ethnicities while in 1,000 Genome project, the Pakistani population is represented by one ethnicity only. The difference in sample size may be another reason for discrepancy.

**Table 6:** CYP2E1 (rs2070672) allele frequencies in 1,000 Genome population.

Population	G	C	GG	GC	CC
AFR	0.852	0.148	0.734	0.236	0.030
AMR	0.889	0.102	0.813	0.170	0.017
EAS	0.733	0.267	0.530	0.407	0.063
EUR	0.974	0.26	0.948	0.52	0
SAS	0.713	0.287	0.513	0.399	0.088
BEB	0.686	0.315	0.488	0.395	0.116
GIH	0.728	0.272	0.524	0.408	0.068
ITU	0.721	0.279	0.520	0.402	0.078
PJL	0.755	0.245	0.594	0.323	0.083
STU	0.672	0.328	0.441	0.461	0.098

**Note:**

AFR, African; AMR, American; EAS, East Asian; EUR, European; SA, South Asian; BEB, Bengali in Bangladesh; GIH, Gujrati Indian in Houston, TX; ITU, Indian Telugu in UK; PJL, Punjabi in Lahore, Pakistan; STU, Sri Lankan Tamil in UK.

Among various ethnicities, Urdu speaking showed the highest frequency of rs2070672 minor allele. Punjabi ethnicity displayed the highest prevalence of the minor allele after Urdu speaking while Baloch people exhibited the lowest frequency of this allele. While comparing genotypes frequencies, Urdu speaking ethnicity showed the lowest frequency of wild type genotype (AA) followed by Punjabi ethnicity. The highest frequency of wild type genotype was exhibited by Baloch ethnicity. Pathan, Baloch and Seraiki ethnicities did not show any homozygous mutant genotype (GG) while Urdu speaking ethnicity displayed the highest frequency of this genotype among the study participants. Studies investigating this polymorphism in the regional populations reveal that the Chinese Uygur population has a low prevalence of this genetic variant at 0.25% (Zhu et al., 2018). Other studies carried out at various geographical locations in China showed much higher frequencies. For example, this variant was found at a frequency of 18.8% in Shantou, 14.1% in Shanghai, 18.8% in Shenyang and 21.9% in Xian (Tang et al., 2010).

Comparison with other regional populations reveal relatively lower frequencies of the minor allele (rs207067) in Pakistani ethnicities. Sri Lankan Tamils in the UK (STU) and Bengali from Bangladesh (BEB) have shown a much higher frequency of minor allele at 0.328 and 0.315 respectively whereas, in Pakistani population, the highest frequency of this allele is reported by Punjabi and Urdu ethnicities at 0.128 and 0.158, respectively. Even Gujrati Indian in Houston (GIH), Punjabi in Lahore (PJL) and Indian Telugu in the UK (ITU) have reported minor allele frequencies that are higher than most Pakistan ethnicities. Although, largely in agreement with regional ethnicities, this relatively low frequency of minor alleles in Pakistani ethnicities might be due to broad geographical locations from which our samples were obtained. For example, PJL data in the 1,000 genome project, was obtained from one center in Lahore while our samples were collected from various centers in Rawalpindi, Islamabad, and Lahore.

The frequency of rs3813865 minor allele (C) in the Pakistani population was similar to the one found in African population. The lowest frequency of the minor allele is reported from Europe (0.026) (Table 7). The highest frequencies of this polymorphism are reported from South and East Asia at 0.287 and 0.267 respectively (Genomes Project Consortium, 2015). The frequency of this genetic variant in the American population is reported at 0.102, which is the second-lowest frequency reported in the 1,000 genome database for this variant. Looking at the genotype frequencies, wild type genotype observed in the Pakistani population was closest to the one reported for the African population. South Asian and East Asian populations are reported to have the lowest frequencies of wild type genotype at 0.513 and 0.530 respectively (Genomes Project Consortium, 2015). The highest frequency of wild type genotype (GG) is reported from European populations at 0.948 with a 0.52 heterozygous genotype (GC). However, European populations are the only ones reported in the 1,000 genome to have no homozygous CC genotype.

**Table 7:** CYP2E1 (rs2070672) allele frequencies in 1,000 Genome population.

Population	A	G	AA	AG	GG
AFR	0.850	0.150	0.735	0.230	0.035
AMR	0.899	0.101	0.816	0.167	0.017
EAS	0.736	0.264	0.534	0.405	0.062
EUR	0.973	0.027	0.946	0.054	0
SAS	0.713	0.287	0.513	0.399	0.088
BEB	0.686	0.314	0.488	0.395	0.116
GIH	0.728	0.272	0. 524	0.408	0.068
ITU	0.721	0.279	0.520	0.402	0.078
PJL	0.755	0.245	0.594	0.323	0.083
STU	0.672	0.328	0.441	0.461	0.098

**Note:**

AFR, African; AMR, American; EAS, East Asian; EUR, European; SA, South Asian; BEB, Bengali in Bangladesh; GIH, Gujrati Indian in Houston, TX; ITU, Indian Telugu in UK; PJL, Punjabi in Lahore, Pakistan; STU, Sri Lankan Tamil in UK.

Comparing rs3813865 polymorphism among various ethnicities revealed that Urdu and Punjabi ethnicities have the highest prevalence of the minor allele (C). The highest major allele frequency was exhibited by Sindhi ethnicity. Pathan, Seraiki and Baloch ethnicity showed frequency of minor allele in the same range but was considerably higher than Sindhi ethnicity. Studying genotype frequencies of various ethnicities showed that Sindhi ethnicity possesses the highest wild type genotype (GG). The lowest wild type genotype was exhibited by Punjabi ethnicity. Pathan, Seraiki, Baloch, and Urdu speaking ethnicities showed an intermediate prevalence of wild type genotype compared to Sindhi and Punjabi ethnicities. Only Sindhi ethnicity did not report any homozygous mutant genotype (CC). Consistent with our findings with rs207067, the frequency of rs3813865 minor allele was found slightly lower in our ethnic populations in comparison with other regional ethnicities. Sri Lankan Tamils in the UK (STU) and Bengali from Bangladesh (BEB) have shown a higher frequency of minor allele at 0.328 and 0.315 respectively whereas, in Pakistani population, the highest frequency of this allele is reported by Punjabi and Urdu ethnicities at 0.185 and 0.188, respectively. Even Gujrati Indian in Houston (GIH), Punjabi in Lahore (PJL) and Indian Telugu in the UK (ITU) have reported minor allele frequencies that are higher than most Pakistan ethnicities. A literature search shows that the frequency of this genetic variant at various geographical locations in China displayed comparable and, in some cases, higher frequencies. For example, this variant was found at a frequency of 18.7% in Shantou, 14% in Shanghai, 23.4% in Shenyang and 22.4% in Xian (Tang et al., 2010).

Limitations of our study include finding out the prevalence of only two SNPs while there are more than 100 SNPs found in *CYP2E1*. However, only a few might alter the enzyme activity or associated with certain diseases. Our methods for the determination of these SNPs also prohibited us from finding novel SNPs in our population. Sequencing all samples could have helped find new SNPs in our population. This would have also helped us finding copy number variants in the *CYP2E1* gene in our population if any. Functional analysis of the CYP2E1 enzyme, containing these SNPs could have helped establish the functional relevance of observed SNPs.

## Conclusions

To our knowledge, this is the first study to report frequencies of *CYP2E1* gene polymorphisms in various ethnicities of the Pakistani population. Genetic information about patients’ *CYP2E1* gene is likely to help physicians prescribe to patients the most suitable and safest drug based on their genetic make-up. We propose further studies with individual drugs metabolized by CYP2E1 to shed more light on genotype phenotype relations. Carrying out enzyme activities of CYP2E1 containing these SNPs would be helpful to establish functional relevance and importance of these SNPs in the Pakistani population.

## Supplemental Information

10.7717/peerj.9721/supp-1 Supplemental Information 1 A representative gel of CYP2E1 (rs3813865) genotyping assay 1.

10.7717/peerj.9721/supp-2 Supplemental Information 2 A representative gel of CYP2E1 (rs2070672) genotyping assay 1.

10.7717/peerj.9721/supp-3 Supplemental Information 3 Polymorphism of CYP2E1 (rs2070672) in the ethnic groups. Shows all subjects examined according to their gender, ethnicity, age, genotype, addiction (eg. smoking), diet (red meat consumption), sedentary life, family history, medication, co-morbidities, and diseases.

10.7717/peerj.9721/supp-4 Supplemental Information 4 Polymorphism of CYP2E1 (rs3813865) in the ethnic groups. Shows all subjects examined according to their gender, ethnicity, age, genotype, addiction (eg. smoking), diet (red meat consumption), sedentary life, family history, medication, co-morbidities, and diseases.

## Additional Information and Declarations

### Data Availability

The following information was supplied regarding data availability: The raw measurements are available in the Supplemental Files.
