# Supplementary figures and images for: Genetic variations in the drug metabolizing enzyme, CYP2E1, among various ethnic populations of Pakistan
Source: PeerJ. 2020 Aug 19;8:e9721. doi: 10.7717/peerj.9721 (PMC7443092; doi:10.7717/peerj.9721)

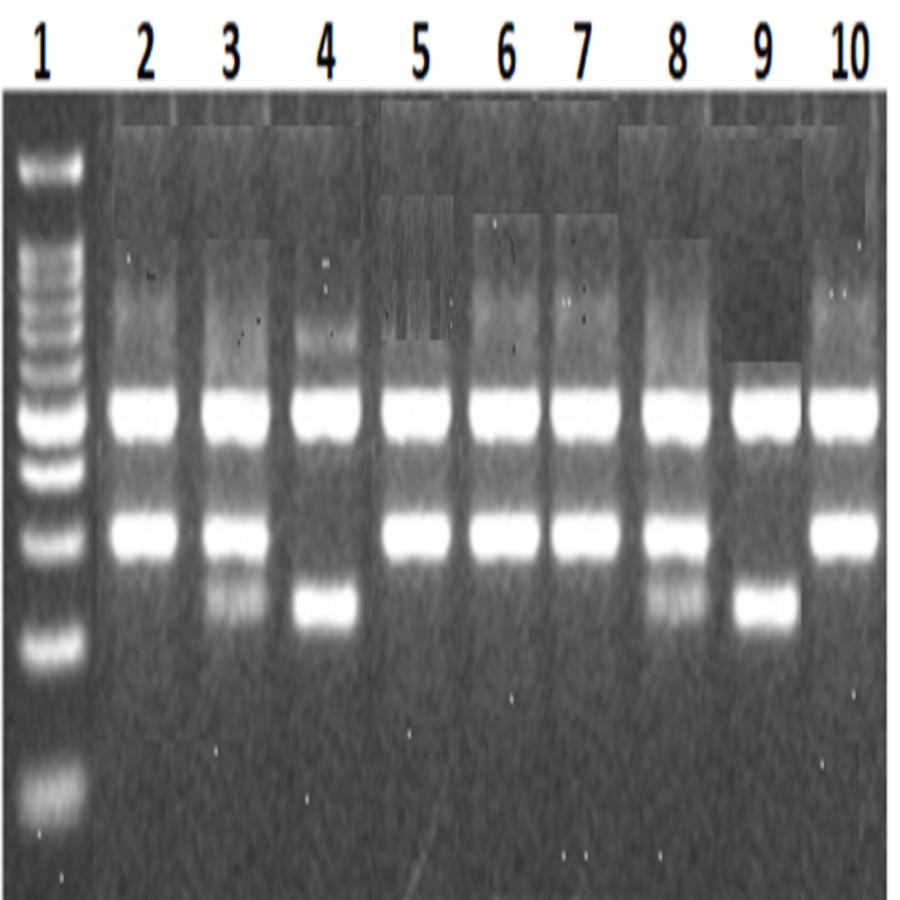

Supplement: Supplemental Information 1 [file peerj-08-9721-s001.jpg]

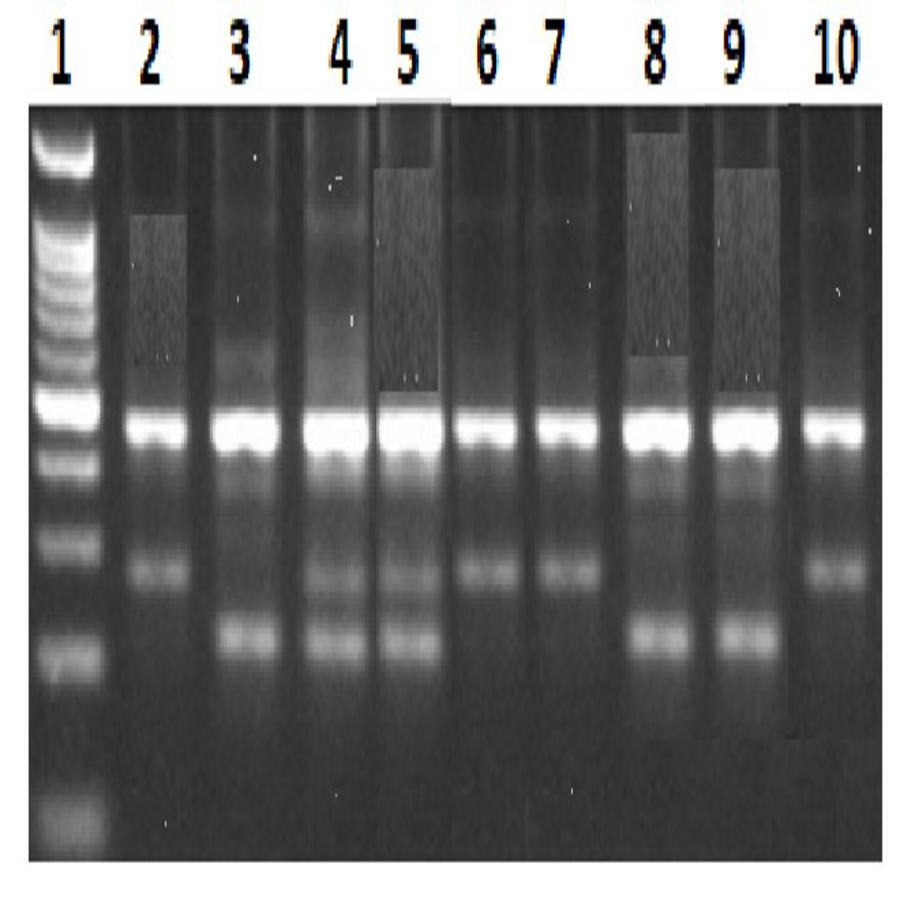

Supplement: Supplemental Information 2 [file peerj-08-9721-s002.jpg]
